# Coefficient Bounds for Some Families of Starlike and Convex Functions of Reciprocal Order

**DOI:** 10.1155/2014/989640

**Published:** 2014-11-24

**Authors:** Muhammad Arif, Maslina Darus, Mohsan Raza, Qaiser Khan

**Affiliations:** ^1^Department of Mathematics, Abdul Wali Khan University Mardan, Mardan, Khyber Pakhtunkhwa 23200, Pakistan; ^2^School of Mathematical Sciences, Faculty of Science and Technology, Universiti Kebangsaan Malaysia, 43600 Bangi, Selangor, Malaysia; ^3^Department of Mathematics, Government College University, Faisalabad 38000, Pakistan

## Abstract

The aim of the present paper is to investigate coefficient estimates, Fekete-Szegő inequality, and upper bound of third Hankel determinant for some families of starlike and convex functions of reciprocal order.

## 1. Introduction

Let *A* denote the class of functions *f*(*z*) which are analytic in the open unit disk *U* = {*z* ∈ *C* : |*z*| < 1} and normalized by

(1) $$\begin{array}{c} f\left(z\right)=z+\overset{\infty }{\underset{k=2}{\sum }}a_{k}z^{k},\;\left(z\in U\right). \end{array}$$

Also let *S* ^*^(*α*) and *K*(*α*) denote the usual classes of starlike and convex functions of order *α*, 0 ≤ *α* < 1, respectively. In 1975, Silverman [1] proved that *f*(*z*) ∈ *S* ^*^(*α*) if it satisfies the condition

(2) $$\begin{array}{c} \left|\frac{zf^{\prime }\left(z\right)}{f\left(z\right)}-1\right|<1-\alpha ,\;\left(z\in U\right). \end{array}$$

Geometrical meaning of inequality (2) is that *zf*′(*z*)/*f*(*z*) maps *U* onto the interior of the circle with center at 1 and radius 1 − *α*.

By *S*_∗_(*α*) and *K*_∗_(*α*), we mean the classes of starlike and convex functions of reciprocal order *α*, 0 ≤ *α* < 1 which are defined, respectively, by

(3) S∗α=fz∈A:Refzzf′z>α,  z∈U,K∗α=fz∈A:Ref′zzf′′z+f′z>α,  z∈U.

Recently in 2008, Nunokawa and his coauthors [2] improved inequality (2) for the class *S*_∗_(*α*) and they proved that, for *f*(*z*) ∈ *S*_∗_(*α*), 0 < *α* < 1/2, if and only if the following inequality holds:

(4) $$\begin{array}{c} \left|\frac{zf^{\prime }\left(z\right)}{f\left(z\right)}-\frac{1}{2\alpha }\right|<\frac{1}{2\alpha },\;\left(z\in U\right). \end{array}$$

In view of these results we now define the following subclass of analytic functions of reciprocal order and investigate its various properties.

**Definition 1 .**

A function *f*(*z*)∈*A* is said to be in the class *L*(*λ*, *γ*), with *γ* ∈ *C*∖{0} and *λ* ∈ [0,1], if it satisfies the inequality

(5) Re1+1γFλzzFλ′z−1>0,

where

(6) $$\begin{array}{c} F_{\lambda }\left(z\right)=\left(1-\lambda \right)f\left(z\right)+\lambda zf^{\prime }\left(z\right). \end{array}$$

**Example 2 .**

Let us define the functions *F*_*λ*_(*z*) by

(7) $$\begin{array}{c} F_{\lambda }\left(z\right)=\frac{z}{{\left(1+\left(2\gamma -1\right)z\right)}^{2\gamma /\left(2\gamma -1\right)}}. \end{array}$$

This implies that

(8) $$\begin{array}{c} \frac{zF_{\lambda }^{\prime }\left(z\right)}{F_{\lambda }\left(z\right)}=\frac{1-z}{1+\left(2\gamma -1\right)z}. \end{array}$$

Hence

(9) $$\begin{array}{c} 1+\frac{1}{\gamma }\left(\frac{F_{\lambda }\left(z\right)}{zF_{\lambda }^{\prime }\left(z\right)}-1\right)=\frac{1+z}{1-z} \end{array}$$

and this further implies that

(10) Re1+1γFλzzFλ′z−1=Re1+z1−z>0, z∈U.

The *q*th Hankel determinant *H*_*q*_(*n*), *q* ≥ 1, *n* ≥ 1, for a function *f*(*z*) ∈ *A* is studied by Noonan and Thomas [3] as

(11) $$\begin{array}{c} H_{q}\left(n\right)=\left|\begin{array}{cccc} a_{n} & a_{n+1} & \cdots & a_{n+q-1}\\ a_{n+1} & a_{n+2} & \cdots & a_{n+q}\\ \vdots & \vdots & \cdots & \vdots \\ a_{n+q-1} & a_{n+q} & \cdots & a_{n+2q-2} \end{array}\right|. \end{array}$$

In literature many authors have studied the determinant *H*_*q*_(*n*). For example, Arif et al. [4, 5] studied the *q*th Hankel determinant for some subclasses of analytic functions. Hankel determinant of exponential polynomials is obtained by Ehrenborg in [6]. The Hankel transform of an integer sequence and some of its properties were discussed by Layman [7]. It is well known that the Fekete-Szegő functional |*a*_3_ − *a*_2_^2^| is *H*_2_(1). Fekete-Szegő then further generalized the estimate |*a*_3_ − *λa*_2_^2^| with *λ* real and *f*(*z*) ∈ *S*. Moreover, we also know that the functional |*a*_2_ *a*_4_ − *a*_3_^2^| is equivalent to *H*_2_(2). The sharp upper bounds of the second Hankel determinant for the familiar classes of starlike and convex functions were studied by Janteng et al. [8]; that is, for *f*(*z*) ∈ *S* ^*^ and *f*(*z*) ∈ *C*, they obtained |*a*_2_ *a*_4_ − *a*_3_^2^| ≤ 1 and 8|*a*_2_ *a*_4_ − *a*_3_^2^| ≤ 1, respectively. In 2007, Babalola [9] considered the third Hankel determinant *H*_3_(1) and obtained the upper bound of the well-known classes of bounded-turning, starlike and convex functions. In 2013 Raza and Malik [10] studied the Hankel third determinant related with lemniscate of Bernoulli. In the present investigation, we study the upper bound of *H*_3_(1) for a subclass of analytic functions of reciprocal order by using Toeplitz determinants.

In this paper we study some useful results including coefficient estimates, Fekete-Szegő inequality, and upper bound of third Hankel determinant for the functions belonging to the class *L*(*λ*, *γ*).

Throughout in this paper we assume that *γ* ∈ *C*∖{0} and *λ* ∈ [0,1] unless otherwise stated.

For our results we will need the following Lemmas.

**Lemma 3 (see [11]).**

If *q*(*z*) is a function with *Req*(*z*) > 0 and is of the form

(12) $$\begin{array}{c} q\left(z\right)=1+c_{1}z+c_{2}z^{2}+\cdots \end{array}$$

then

(13) $$\begin{array}{c} \left|c_{n}\right|\leq 2,\;for\;\;n\geq 1. \end{array}$$

**Lemma 4 (see [12]).**

If *q*(*z*) is of the form (12) with positive real part, then the following sharp estimate holds:

(14) $$\begin{array}{c} \left|c_{2}-\nu c_{1}^{2}\right|\leq 2\max\left\{1,\left|2\nu -1\right|\right\},\;for\;all\;\nu \in C. \end{array}$$

**Lemma 5 (see [13]).**

If *q*(*z*) is of the form (12) with positive real part, then

(15) 2c2=c12+x4−c12,4c3=c13+24−c12c1x−c14−c12x2 +24−c121−x2z,

for some *x*, *z* with |*x*| ≤ 1 and |*z*| ≤ 1.

## 2. Some Properties of the Class *L*(*λ*, *γ*)

**Theorem 6 .**

Let *f*(*z*) ∈ *L*(*λ*, *γ*). Then

(16) $$\begin{array}{c} \left|a_{2}\right|\leq \frac{2|\gamma |}{1+\lambda }, \end{array}$$

and for all *n* = 3,4, 5,…

(17) $$\begin{array}{c} \left|a_{n}\right|\leq \frac{2|\gamma |}{\left(n-1\right)\left(1+\lambda \left(n-1\right)\right)}\overset{n-1}{\underset{k=2}{\prod }}\left(1+\frac{2\left|\gamma \right|k}{k-1}\right). \end{array}$$

**Proof**

Let us define the function *q*(*z*) by

(18) $$\begin{array}{c} q\left(z\right)=1+\frac{1}{\gamma }\left(\frac{F_{\lambda }\left(z\right)}{zF_{\lambda }^{\prime }\left(z\right)}-1\right), \end{array}$$

where *F*_*λ*_(*z*) is given by (6) with

(19) $$\begin{array}{c} F_{\lambda }\left(z\right)=z+\overset{\infty }{\underset{k=2}{\sum }}\left[1+\lambda \left(k-1\right)\right]a_{k}z^{k}, \end{array}$$

and *q*(*z*) is analytic in *U* with *q*(0) = 1, *Req*(*z*) > 0.

Now using (1) and (12), we have

(20) $$\begin{array}{c} z+\overset{\infty }{\underset{k=2}{\sum }}A_{k}z^{k}=\left[1+\gamma \left(\overset{\infty }{\underset{k=1}{\sum }}c_{k}z^{k}\right)\right]\left(z+\overset{\infty }{\underset{k=2}{\sum }}kA_{k}z^{k}\right), \end{array}$$

where

(21) $$\begin{array}{c} A_{k}=\left[1+\lambda \left(k-1\right)\right]a_{k}. \end{array}$$

Comparing coefficient of like power of *z* ^*n*^, we obtain

(22) $$\begin{array}{c} \left(1-n\right)A_{n}=\gamma \left\{c_{n-1}+2A_{2}c_{n-2}+\cdots +\left(n-1\right)A_{n-1}c_{1}\right\}. \end{array}$$

Using triangle inequality and Lemma 3, we get

(23) $$\begin{array}{c} \left|\left(1-n\right)A_{n}\right|\leq 2\left|\gamma \right|\left\{1+2\left|A_{2}\right|+\cdots +\left(n-1\right)\left|A_{n-1}\right|\right\}. \end{array}$$

For *n* = 2 and *n* = 3 in (23), we easily obtain that

(24) $$\begin{array}{c} \left|a_{2}\right|\leq \frac{2\left|\gamma \right|}{1+\lambda },\;\;\left|a_{3}\right|\leq \frac{\left|\gamma \right|\left(1+4\left|\gamma \right|\right)}{1+2\lambda }. \end{array}$$

Making *n* = 4 in (23), we see that

(25) $$\begin{array}{c} 2\left|A_{3}\right|\leq 2\left|\gamma \right|\left(1+2\left|A_{2}\right|\right)\leq 2\left|\gamma \right|\left\{1+\frac{4\left|\gamma \right|\left(1+2\lambda \right)}{1+\lambda }\right\}; \end{array}$$

equivalently, we have

(26) $$\begin{array}{c} \left|a_{4}\right|\leq \frac{2\left|\gamma \right|\left(1+3\left|\gamma \right|\right)\left(1+4\left|\gamma \right|\right)}{3\left(1+3\lambda \right)}. \end{array}$$

Using the principal of mathematical induction, we obtain

(27) $$\begin{array}{c} \left|A_{n}\right|\leq \frac{2\left|\gamma \right|}{\left(n-1\right)}\overset{n-1}{\underset{k=2}{\prod }}\left(1+\frac{2\left|\gamma \right|k}{k-1}\right). \end{array}$$

Now from the use of relation (21), we obtain the required result.

If we take *λ* = 0 and *γ* = 1 − *α*, we get the following result.

**Corollary 7 (see [14]).**

Let *f*(*z*) ∈ *S*_∗_(*α*). Then, for *n* = 3,4, 5,…, one has

(28) $$\begin{array}{c} \left|a_{n}\right|\leq \frac{2\left(1-\alpha \right)}{\left(n-1\right)}\overset{n-1}{\underset{k=2}{\prod }}\left(1+\frac{2\left(1-\alpha \right)k}{k-1}\right), \end{array}$$

with |*a*_2_| ≤ 2(1 − *α*).

Making *λ* = 1 and *γ* = 1 − *α*, we get the following result.

**Corollary 8 (see [14]).**

Let *f*(*z*) ∈ *K*_∗_(*α*). Then, for *n* = 3,4, 5,…, one has

(29) $$\begin{array}{c} \left|a_{n}\right|\leq \frac{2\left(1-\alpha \right)}{n\left(n-1\right)}\overset{n-1}{\underset{k=2}{\prod }}\left(1+\frac{2\left(1-\alpha \right)k}{k-1}\right) \end{array}$$

with |*a*_2_| ≤ (1 − *α*).

**Theorem 9 .**

Let *f*(*z*) ∈ *L*(*λ*, *γ*) and be of the form (1). Then

(30) $$\begin{array}{c} \left|a_{3}-\mu a_{2}^{2}\right|\leq \frac{\left|\gamma \right|}{\left(1+2\lambda \right)}\max\left\{1,\left|2\nu -1\right|\right\}, \end{array}$$

where

(31) $$\begin{array}{c} \nu =2\gamma \left(1+2\lambda \right)\left(\frac{1}{1+2\lambda }-\frac{\mu }{{\left(1+\lambda \right)}^{2}}\right). \end{array}$$

**Proof**

Let *f*(*z*) ∈ *L*(*λ*, *γ*). Then from (22) we have

(32) $$\begin{array}{c} a_{2}=\frac{-\gamma c_{1}}{\left(1+\lambda \right)},\;\;a_{3}=\frac{-\gamma }{2\left(1+2\lambda \right)}\left(c_{2}-2\gamma c_{1}^{2}\right). \end{array}$$

We now consider

(33) a3−μa22 =γ21+2λc2−2γ1+2λ11+2λ−μ1+λ2c12.

Using Lemma 4, we obtain

(34) $$\begin{array}{c} \left|a_{3}-\mu a_{2}^{2}\right|\leq \frac{\left|\gamma \right|}{\left(1+2\lambda \right)}\max\left\{1,\left|2\nu -1\right|\right\}, \end{array}$$

where *ν* is given by (31).

Putting *μ* = 1, we obtain the following result.

**Corollary 10 .**

Let *f*(*z*) ∈ *L*(*λ*, *γ*). Then

(35) $$\begin{array}{c} \left|a_{3}-a_{2}^{2}\right|\leq \frac{\left|\gamma \right|}{\left(1+2\lambda \right)}. \end{array}$$

**Theorem 11 .**

Let *f*(*z*) ∈ *L*(*λ*, *γ*) and be of the form (1). Then

(36) a2a4−a32 ≤7+28λ+25λ2+41+4λ+10λ2γ+48λ2γ231+2λ21+4λ+3λ2  ×γ2.

**Proof**

Let *f*(*z*) ∈ *L*(*λ*, *γ*). Then, from (22), we have

(37) a2=−γc11+λ,a3=−γ21+2λc2−2γc12,a4=−γ31+3λc3−7γ2c1c2+3γ2c13.

Consider

(38) a2a4−a32 =γ2121+λ1+2λ21+3λ   ×41+2λ2c1c3−2γ1+4λ+10λ2c12c2    +12γ2λ2c14−31+4λ+3λ2c22γ2121+λ1+2λ21+3λ.

Now using values of *c*_2_ and *c*_3_ from Lemma 5, we obtain

(39) a2a4−a32=γ2121+λ1+2λ21+3λ ×341+2λ2−γ1+4λ+10λ2  +12γ2λ2−341+4λ+3λ2c14  +21+2λ2−γ1+4λ+10λ2−321+4λ+3λ2  ×4−c12c12x  −1+2λ2c12+341+4λ+3λ24−c12  ×4−c12x2+2c11+2λ24−c121−x2z34.

Applying triangle inequality and replacing *c*_1_ by *c*, |*x*| by *ρ*, and |*z*| by 1, we get

(40) a2a4−a32≤γ2121+λ1+2λ21+3λ ×341+2λ2+γ1+4λ+10λ2    +12γ2λ2+341+4λ+3λ2c4 +21+2λ2+γ1+4λ+10λ2+321+4λ+3λ2 ×4−c2c2ρ +1+2λ2c2+341+4λ+3λ24−c2 ×4−c2ρ2+2c1+2λ24−c21−ρ2341+2λ2+γ1+4λ+10λ2=Fc,ρ.

Differentiating with respect to *ρ*, we get

(41) ∂Fc,ρ∂ρ =γ2121+λ1+2λ21+3λ  ×3221+2λ2+γ1+4λ+10λ2    +321+4λ+3λ24−c2c2    +21+2λ2c2+321+4λ+3λ24−c2    ×4−c2ρ−4c1+2λ24−c2ρ32.

Now since ∂*F*(*c*, *ρ*)/∂*ρ* > 0 for *c* ∈ [0,2] and *ρ* ∈ [0,1], maximum of *F*(*c*, *ρ*) will exist at *ρ* = 1 and let *F*(*c*, 1) = *G*(*c*). Then

(42) Gc=γ2121+λ1+2λ21+3λ ×341+2λ2+γ1+4λ+10λ2   +12γ2λ2+341+4λ+3λ2c4   +3421+2λ2+γ1+4λ+10λ2    +321+4λ+3λ24−c2c2   +1+2λ2c2+341+4λ+3λ24−c2   ×4−c234.

Now by differentiating with respect to *c*, we obtain

(43) G′c=γ2121+λ1+2λ21+3λ ×4341+2λ2+γ1+4λ+10λ2    +12γ2λ2+341+4λ+3λ2c3   +3421+2λ2+γ1+4λ+10λ2    +321+4λ+3λ28c−4c3   +1+2λ28c−4c3    −31+4λ+3λ24c−c334.

Since ∂*G*(*c*)/∂*c* > 0 for *c* ∈ [0,2], *G*(*c*) has a maximum value at *c* = 2 and hence

(44) a2a4−a32 ≤7+28λ+25λ2+41+4λ+10λ2γ+48λ2γ231+2λ21+4λ+3λ2  ×γ2.

**Theorem 12 .**

Let *f*(*z*) ∈ *L*(*λ*, *γ*) and be of the form (1). Then

(45) $$\begin{array}{c} \left|a_{2}a_{3}-a_{4}\right|\leq \frac{2\left|\gamma \right|\left[\left(4\left|\gamma \right|+1\right)\left(\left(6\left|\gamma \right|+2\right)\lambda^{2}+\left(3\lambda +1\right)\right)\right]}{3\left(1+\lambda \right)\left(1+2\lambda \right)\left(1+3\lambda \right)}. \end{array}$$

**Proof**

From (37), we can write

(46) a2a3−a4=12γ2λ2c13−2γ(2+6λ+7λ2)c1c2+21+3λ+2λ2c361+λ1+2λ1+3λ ×γ.

Using Lemma 5 for the values of *c*_2_ and *c*_3_, we have

(47) a2a3−a4 =γ61+λ1+2λ1+3λ  ×12(4γ−1)(2(3γ−1)λ2−3λ+1)c13   +1+3λ+2λ2−γ2+6λ+7λ2   ×(4−c12)c1x   −121+3λ+2λ2(4−c12)c1x2   +1+3λ+2λ2(4−c12)1−x2z12.

Applying triangle inequality and then putting |*z*| = 1, |*x*| = *ρ*, and *c*_1_ = *c*, we have

(48) a2a3−a4 ≤γ61+λ1+2λ1+3λ  ×4γ+16γ+2λ2+3λ+1c32    +1+3λ+2λ2+γ2+6λ+7λ2    ×4−c2cρ+121+3λ+2λ24−c2cρ2    +1+3λ+2λ24−c21−ρ2c32=Fc,ρ.

Now by using the same procedure as we did in the proof of Theorem 11, we obtain the required result.

**Theorem 13 .**

If *f*(*z*) ∈ *L*(*λ*, *γ*) and is of the form (1), then

(49) H31 ≤7+28λ+25λ2+41+4λ+10λ2γ+48λ2γ231+2λ31+4λ+3λ2  ×γ31+4γ  +44γ+123γ+12λ2+3λ+191+λ1+2λ1+3λ2  ×γ21+3γ  +1+4γ1+3γ3+8γγ261+2λ1+4λ.

**Proof**

Since

(50) $$\begin{array}{c} \left|H_{3}\left(1\right)\right|\leq \left|a_{3}\right|\left|a_{2}a_{4}-a_{3}^{2}\right|+\left|a_{4}\right|\left|a_{2}a_{3}-a_{4}\right|+\left|a_{5}\right|\left|a_{1}a_{3}-a_{2}^{2}\right|, \end{array}$$

using Theorem 6, Corollary 10, and Theorems 11 and 12, we have

(51) H31≤γ1+4γ1+2λ ×7+28λ+25λ2+41+4λ+10λ2γ+48λ2γ231+2λ21+4λ+3λ2 ×γ2+2γ1+4γ1+3γ31+3λ ×2γ4γ+13γ+12λ2+3λ+131+λ1+2λ1+3λ +γ1+4γ1+3γ3+8γ61+4λγ1+2λ=7+28λ+25λ2+41+4λ+10λ2γ+48λ2γ231+2λ31+4λ+3λ2 ×γ31+4γ +44γ+123γ+12λ2+3λ+191+λ1+2λ1+3λ2 ×γ21+3γ+1+4γ1+3γ3+8γγ261+2λ1+4λ.

This completes the proof of this result.
